# Actividad inhibitoria del extracto etanólico del *Cyperus Rotundus* procedente de la región de Cajamarca (provincia de Contumazá) en una cepa estandarizada de S*treptococcus mutans* (ATCC ^®^ 25175 ^TM^ )

**DOI:** 10.21142/2523-2754-1001-2022-093

**Published:** 2022-03-30

**Authors:** Rosita Belén Bazán Aliaga, Óscar Reátegui Arévalo, Luz Verónica Solórzano Espinoza, Juan Antonio Castro Arredondo, Víctor Elmo Miranda García, Elba Estefanía Martínez Cadillo, Ángela Quispe-Salcedo

**Affiliations:** 1 Carrera de Estomatología, Universidad Científica del Sur. Lima, Perú. 100030721@cientifica.edu.pe, jcastroar@cientifica.edu.pe, emartinez@cientifica.edu.pe, aquispesa@cientifica.edu.pe Universidad Científica del Sur Carrera de Estomatología Universidad Científica del Sur Lima Peru 100030721@cientifica.edu.pe jcastroar@cientifica.edu.pe emartinez@cientifica.edu.pe aquispesa@cientifica.edu.pe; 2 Dirección General de Investigación, Desarrollo e Innovación (DGIDI) de la Universidad Científica del Sur. Lima, Perú. oreategui@cientifica.edu.pe Universidad Científica del Sur Dirección General de Investigación, Desarrollo e Innovación (DGIDI) Universidad Científica del Sur Lima Peru oreategui@cientifica.edu.pe; 3 Sede Villa. Universidad Científica del Sur. Lima, Perú. luzveronicasolorzano@gmail.com Universidad Científica del Sur Sede Villa Universidad Científica del Sur Lima Peru luzveronicasolorzano@gmail.com; 4 Laboratorio de Química y Bioquímica de Productos Naturales, Universidad Científica del Sur. Lima, Perú. vmirandag@cientifica.edu.pe Universidad Científica del Sur Laboratorio de Química y Bioquímica de Productos Naturales Universidad Científica del Sur Lima Peru vmirandag@cientifica.edu.pe

**Keywords:** Streptococcus mutans, efecto inhibitorio, extracto etanólico, Cyperus rotundus, Streptococcus mutans, inhibitory effect, ethanolic extract, Cyperus rotundus

## Abstract

**Objetivo:**

Determinar *in vitro* la actividad inhibitoria del extracto etanólico del *Cyperus rotundus* (Contumazá, Cajamarca) frente a una cepa estandarizada de *Streptococcus mutans* (ATCC®25175^TM^).

**Materiales y métodos:**

El presente estudio fue de tipo experimental *in vitro*, el cual consistió en determinar el efecto inhibitorio de tres concentraciones del extracto etanólico de *Cyperus rotundus*: 250 mg/ml, 500 mg/ml y 1000 mg/ml frente a *Streptococcus mutans* (ATCC®25175^TM^). Se realizaron 10 pruebas para cada concentración del extracto, y se tuvo como control positivo la clorhexidina al 0,12% y como control negativo, al DMSO al 10%. Para evaluar el efecto inhibitorio, se utilizó el método de difusión en discos o prueba de Kirby-Bauer, y se realizó la lectura de las placas de cultivo a las 48 horas posteriores a la siembra.

**Resultados:**

Ninguna de las tres concentraciones del extracto etanólico de *Cyperus rotundus* presentó efecto inhibitorio para la cepa de *Streptococcus mutans*; sin embargo, tras el control positivo, la clorhexidina presentó halos de inhibición de 14,43 mm ± 1,23 mm, a las 48 horas de incubación.

**Conclusiones:** Se determinó que el extracto etanólico del *Cyperus rotundus* no es capaz de inhibir el crecimiento de *Streptococcus mutans*. Se recomienda profundizar en el análisis químico de los componentes de esta planta y explorar otros metodos de extracción para verificar su acción bacteriostática en otros microorganismos orales y no orales.

## Abstract (en)

**Objective:**

The purpose of this study was to determine in vitro the inhibitory activity of the ethanolic extract of Cyperus rotundus (Cajamarca - Contumazá) against a standardize strain of *Streptococcus mutans* (ATCC®25175™).

**Materials and methods:**

This study was an experimental in vitro study, which consisted of determining the inhibitory effect of three concentrations of the ethanolic extract of *Cyperus rotundus*: 250 mg/ml, 500 mg/ml, and 1000 mg/ml against a strains of *Streptococcus mutans* (ATCC®25175™). Ten tests were performed for each concentration, having 0.12% chlorhexidine as a positive control for *Streptococcus mutans* plates and 10% DMSO as a negative control. To evaluate the inhibitory effect, the disk diffusion method or Kirby-Bauer test was used, reading the results at 48 hours after initial sowing.

**Results:**

None of the three concentrations of the ethanolic extract of *Cyperus rotundus* demosntrated inhibitory effects on the *Streptococcus mutans* strain; however, the positive control, chlorhexidine, clearly showed inhibition halos of 14.43 mm ± 1.23 mm after 48 hours of incubation.

**Conclusions:**

The ethanolic extract of *Cyperus rotundus* did not inhibit the growth of *Streptococcus mutans*. It is recommended to deepen the chemical analysis of the components of this plant and explore other extraction methods to verify its bacteriostatic action versus other oral and non-oral microorganisms.

## INTRODUCCIÓN

El Perú es uno de los países con mayor biodiversidad del planeta debido a su riqueza animal y vegetal ^1^. Popularmente, se emplean 1400 plantas con fines medicinales ^2^. Debido a que un gran porcentaje de la población peruana tiene un acceso limitado a los sistemas de salud y las medicinas industrializadas, las plantas medicinales siguen siendo usadas en la actualidad para la cura de diversas enfermedades, a partir de las creencias o experiencias previas obtenidas a lo largo de la historia. Por tanto, resulta de importancia para la población investigar aquellos productos naturales de uso convencional y fácil acceso para el tratamiento de enfermedades, a fin de enumerar sus propiedades fitoterapéuticas y descartar todo riesgo de toxicidad por su consumo ^2^^-^^5^. 

Estudios realizados sobre el *Cyperus rotundus* determinan que sus propiedades antipiréticas, antiinflamatorias, antifúngicas, antomicrobianas, antimutagénicas, analgésicas e, inclusive, anticariogénicas se deben a sus componentes como alcaloides, compuestos fenólicos, glucósidos, péptidos, ácidos orgánicos y esteroides ^6^^-^^8^. A pesar de sus numerosos beneficios y su abundancia en América Latina ^9^, el número de investigaciones relacionadas con su utilidad para el tratamiento de afecciones orales es bastante reducido en comparación con otras plantas de uso medicinal. 

La caries dental es una enfermedad infecciosa de naturaleza dinámica y multifactorial ocasionada principalmente por la bacteria *Streptococcus mutans*^10^^-^^12^, la cual, en conjunto con otros microorganismos concomitantes, como hongos orales, potencian su patogenicidad ocasionando problemas mayores en las estructuras dentarias y la cavidad oral. ^11^^)(^^13^^-^^15^

Actualmente, la clorhexidina es el antiséptico más utilizado por su eficacia contra las bacterias grampositivas, gramnegativas, anaerobios facultativos, aerobios y, con menor eficacia, frente a levaduras y hongos. En el área odontológica, se ha demostrado que inhibe la adhesión de los microorganismos a la superficie dentaria, lo que impide la formación de la biopelícula dental, por ello se utiliza como coadyuvante en el tratamiento de enfermedades como caries dental, periodontitis, gingivitis y candidiasis oral ^16^^-^^18^. 

Por otro lado, diversas investigaciones han demostrado la efectividad del extracto etanólico del *Cyperus rotundus*, que favorece la inhibición del crecimiento bacteriano, así como la disminución de la producción de ácidos, la adherencia y el impedimento de síntesis de glucanos del *Streptococcus mutans*^7^^,^^19^. Con respecto a los estudios previos, se ha demostrado su baja toxicidad ^7^, lo que lo podría respaldar su uso con fines terapéuticos. A pesar de los diversos estudios encontrados que evidencian las propiedades beneficiosas del extracto etanólico del *Cyperus rotundus*, no se han realizado estudios que evalúen el efecto inhibitorio que la variedad peruana posee frente a microorganismos orales. Por tanto, el propósito de esta investigación fue determinar la actividad inhibitoria del extracto etanólico del *Cyperus rotundus* (Contumazá, Cajamarca) sobre el crecimiento de una de las bacterias más importantes en el establecimiento de la enfermedad de caries dental, el *Streptococcus mutans*, a fin de establecer los beneficios de esta planta medicinal y su impacto en el área odontológica.

## MATERIALES Y MÉTODOS

El presente es un estudio del tipo experimental, *in vitro*, realizado en el Laboratorio de Química y Bioquímica de Productos Naturales, ubicado en la Facultad de Ciencias de la Salud de la Universidad Científica del Sur, sobre una cepa estándar de *Streptococcus mutans* (ATCC®25175^TM^). El proyecto fue presentado para su revisión y aprobado por el Comité Institucional de Ética en Investigación de la Universidad Científica del Sur, con código 014-2019-PRO99.

Se utilizó la fórmula de comparación de medias a un nivel de confianza del 95% y con un margen de error del 5%, a partir de los resultados de una prueba piloto. Esto dio como resultado 10 placas Petri para la prueba frente a *Streptococcus mutans* (ATCC®25175^TM^). Los criterios de inclusión fueron placas Petri con crecimiento verificado de la cepa de *Streptococcus mutans*, mientras que los criterios de exclusión fueron las cepas contaminadas con otras bacterias u hongos, y que no mostraron crecimiento del microorganismo seleccionado.

Las sustancias experimentales usadas fueron el extracto etanólico del *Cyperus rotundus* (EECR) a 250, 500 y 1000 mg/ml, lo que se comparó con la clorhexidina al 0,12% (CHX), como control positivo, y el DMSO al 10%, como control negativo. 

### Obtención del extracto etanólico de *Cyperus rotundus*

**Figura 1:**
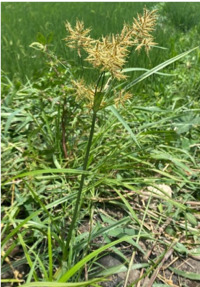
Planta de *Cyperus rotundus* original de la provincia de Contumazá, región Cajamarca

Los EECR se obtuvieron a partir de la raíz y tubérculos del *Cyperus rotundus*, proveniente de la ciudad de Cajamarca, provincia de Contumazá (Figura 1), y se procedió a su identificación y reconocimiento en el Herbario San Marcos del Museo de Historia Natural de la Universidad Nacional Mayor de San Marcos (Lima, Perú).

Las raíces y tubérculos seleccionados de las plantas de *Cyperus rotundus* fueron lavadas, secadas y colocadas en una estufa (MMM Group, Alemania), a una temperatura de 40 ºC por dos días consecutivos. Seguidamente, se realizó la molienda utilizando una licuadora (Oster, EE. UU.) de 2 caballos de fuerza, para luego pesar el producto en balanza digital (Sartorius, Alemania) y colarlo, a fin de obtener una muestra más fina. La molienda se colocó en un frasco ámbar los cuales se llenaron con etanol al 70% (1:6 g/ml). Los frascos fueron colocados sobre un agitador digital (Thermo Fisher Scientific, EE. UU.) por dos días para realizar la excitación de sus componentes. El producto fue homogenizado (Homogenizer-Glas-Col®, EE. UU.), filtrado con papel Whatman n.o 1 en una campana extractora de gases (Labconco, México) y colocado en un frasco color ámbar con cubierta de aluminio, en condiciones de refrigeración (5 ºC), por dos días. Seguidamente, se procedió a utilizar el rotavapor (presión: -1 milibar, T: 48 ºC, rpm: 18) y colocar la muestra en placas Petri para ponerlas en la estufa, a 40 ºC, por dos días. La muestra en seco se colocó en un frasco ámbar y se obtuvo como peso final 15,3305 g, los cuales fueron disueltos en DMSO al 10% y se almacenaron en condiciones de refrigeración (5 ºC), cubiertos con papel aluminio. Finalmente, y a partor del producto obtenido, se realizaron las diluciones respectivas para obtener tres diferentes concentraciones del EECR: 250 mg/ml, 500 mg/ml, y 1000 mg/ml.

### Procedimientos microbiológicos

Se emplearon cepas de *Streptococcus mutans* (ATCC®25175^TM^), adquiridas a través de la empresa GenLab del Perú S. A. C. Se las reactivó en agar mitis salivarius para luego colocarla en microanaerobiosis empleando la jarra GasPak, de acuerdo con el método de vela en extinción, durante 4 días, y una incubación posterior a 37 °C. 

La susceptibilidad del *Streptococcus mutans* frente al EECR, a diferentes concentraciones (250, 500 y 1000 mg/ml) fue determinada usando la prueba de difusión con discos (test de Kirby-Bauer). En primer lugar, se preparó un caldo tripticasa soya (TSB), el cual consistió en 60 ml de H2O destilada y 2 gramos de (TSB), para luego llevarlo al autoclave a 121 ºC por 15 minutos. Se procedió a suspender las cepas de cada microorganismo en tubos de ensayo con el caldo preparado hasta alcanzar la turbidez de 0,5 de la escala de Mc Farland, que fue medida por el espectrofotómetro, para finalmente ser incubados por dos horas a 37 ºC. Posteriormente, se estriaron las cepas de cada microorganismo por medio de siembra por diseminación en agar Müeller Hinton y se incubaron por 2 horas a 37 ºC. 

Para determinar el efecto antibacteriano se colocaron los discos de papel filtro de 5 mm de diámetro y 0,02 mm de espesor, sobre los cuales se agregó 10 µl de cada concentración del extracto etanólico del *Cyperus rotundus* (250, 500 y 1000 mg/ml), clorhexidina al 0,12%, como control positivo (+), y DMSO al 10%, como control negativo (-). Los discos de papel filtro embebidos con cada concentracion del extracto, los controles negativos y positivos fueron ubicados con una pinza estéril sobre los cultivos de las placas Petri anteriormente preparadas, fueron incubadas en microanaerobiosis empleando la jarra de GasPak, siguiendo el método de vela en extinción para que se obtenga un ambiente aproximadamente del 5 a 10% de CO2, a 37 ºC durante 48 horas. Al término del periodo de incubación, se procedió a realizar la lectura de resultados por medio de la inspección visual. El registro el diámetro de los halos de inhibición del crecimiento de *Streptococcus mutans* se realizó con una regla calibrada (Vernier), la cual determinó cuantitativamente el efecto inhibitorio *in vitro*, de acuerdo con el diámetro del halo de inhibición.

### Recolección y análisis de los datos

Los datos fueron colocados en una ficha específicamente creada para este estudio. El análisis estadístico fue realizado usando un *software* especializado (Minitab 19, EE. UU.). El plan de análisis buscó comparar la media entre los grupos estudiados. Para esto se realizó el análisis descriptivo, el cual consistió en detallar la tendencia central (media y mediana) y las medidas de dispersión del halo de inhibición (desviación estándar y varianza), para cada uno de los grupos con los tratamientos utilizados. Seguidamente se evaluó la normalidad con la prueba de Shapiro-Wilk. Una vez comprobada la normalidad de los datos, se utilizó la prueba de ANOVA, y para encontrar la diferencia entre los datos obtenidos se utilizó la prueba HSD de Tukey con un nivel de significancia del 5%.

## RESULTADOS

**Figura 2:**
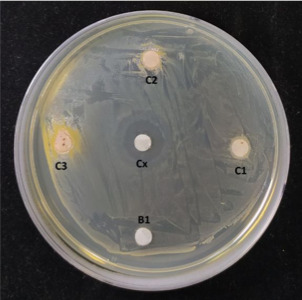
Halos de inhibición a las 48 horas frente a *S. mutans*. C1: EECR 250 mg/ml. C2: EECR 500 mg/ml. C3: EECR 1000 mg/ml. C: CHX 0,12%. B1: DMSO 10%.

No se encontró efecto inhibitorio de las sustancias experimentales para *Streptococcus mutans*. Los EECR a 250, 500 y 1000 mg/ml no mostraron halos de inhibición del crecimiento bacteriano; sin embargo, la clorhexidina al 0,12% presentó un halo de inhibición de 14,4 mm a las 48 horas de lectura (Figura 2, Tabla 1). 

**Tabla 1:** Evaluación descriptiva de la actividad inhibitoria del extracto etanólico de *Cyperus rotundus* a diferentes concentraciones frente a *Streptococcus mutans* a las 48 horas

Sustancias experimentales	N.º de repeticiones	Halo de inhibición (diámetro en mm)		
		Media ± D. S.	Varianza	Mediana
Extracto etanólico del *Cyperus rotundus* 250 mg/ml	10	0	0	0
Extracto etanólico del *Cyperus rotundus* 500 mg/ml	10	0	0	0
Extracto etanólico del *Cyperus rotundus* 1000 mg/ml	10	0	0	0
DMSO 10%	10	0	0	0
Clorhexidina 0,12%	10	14,43 ± 1,23	1,51	14,60

Para la prueba de normalidad se usó la prueba de Shapiro-Wilk, en la cual se mostró que para *Streptococcus mutans*, a las 48 horas, el valor de p fue mayor a 0,1 (p > 0,1), lo que demuestra la normalidad de los datos encontrados para clorhexidina a un nivel de significancia del 5%. Esta prueba de normalidad no se pudo realizar con los datos de las tres concentraciones del EECR, ya que sus valores fueron nulos.

La significancia de los datos obtenidos de los EECR para *Streptococcus mutans* se comparó con la prueba de Anova de un factor. El efecto inhibitorio del tratamiento de clorhexidina para *Streptococcus mutans* con respecto a las concentraciones del EECR poseen diferencias significativas (p < 0,001). Esto se realizó con la prueba HSD de Tukey empleando un nivel de significancia del 5% (Tabla 2).

**Tabla 2:** Comparación de la actividad inhibitoria del extracto etanólico de *Cyperus rotundus* a diferentes concentraciones frente a *Streptococcus mutans* a las 48 horas

SUSTANCIAS EXPERIMENTALES	Media ± D. S. Halo de inhibición (diámetro en mm)	P
Extracto etanólico del *Cyperus rotundus* 250 mg/ml	0	< 0,001*
Extracto etanólico del *Cyperus rotundus* 500 mg/ml	0	
Extracto etanólico del *Cyperus rotundus* 1000 mg/ml	0	
DMSO 10%	0	
Clorhexidina 0,12%	14,43 ± 1,23	

* Nivel de significancia p ≤ 0,05. Prueba de Anova de un factor y HSD de Tukey

## DISCUSIÓN

El presente trabajo de investigación constituye el primer reporte sobre las propiedades de la variedad peruana del *Cyperus rotundus* frente a uno de los microorganismos orales más influyentes y determinantes en el establecimiento de lesiones por caries dental, el *Streptococcus mutans*.

Nagarajan *et al*. ^20^ describen al *Cyperus rotundus* como una de las plantas más usadas en la medicina china tradicional, de la cual se han formulado más de 500 patentes para su uso. Es con base en esta y otras publicaciones, las cuales demuestran su efectividad antibacteriana y antifúngica, ^6^^-^^8^^,^^21^^,^^22^, que surge el interés de investigar las propiedades de la variedad peruana del *Cyperus rotundus* en el área odontológica. 

Khojaste *et al*. ^23^ encontraron que el extracto etanólico del tubérculo del *Cyperus rotundus* mostró un efecto inhibitorio para el *Streptococcus mutans*, al presentar un halo de inhibición de 19 mm a una concentración de 500 mg/ml utilizando el método de pozos en agar; además, comparan el extracto etanólico, acuoso y aceite esencial, siendo el más eficiente el extracto etanólico. La literatura muestra muchos más estudios del *Cyperus rotundus* realizados a partir de un extracto etanólico ^7^^,^^8^^,^^19^^,^^24^, razón por la cual también se decidió optar por esta metodología. El extracto etanólico elaborado para este estudio fue extraído de los bulbos de las raíces, según la metodología de Yu *et al*. ^7^ y Kabbashi *et al*. ^22^, con algunas modificaciones, lo cual constituyó otra razón por la cual se decidió realizar el extracto etanólico, ya que, para producir un aceite esencial, se necesitaría mucha más cantidad de la muestra ^23^.

El análisis microbiológico se llevó a cabo mediante la técnica de difusión en discos (Kirby-Bauer), la cual ha sido empleada por la gran mayoría de autores en este tema ^1^^,^^11^^,^^19^^,^^25^. Esta técnica es frecuentemente utilizada y se basa en el uso de discos de papel esterilizados empapados con el extracto experimental o la solución control. Sin embargo, Montero *et al*. ^26^, en su estudio, informan que el método de pozos modificados de agar es mejor, ya que muestra mayor sensibilidad para el uso de la sustancia contra las bacterias que se desea estudiar.

Haghgoo *et al*. ^19^ encontraron que la concentración inhibitoria mínima (CIM) y la concentración bactericida mínima (CBM) del extracto etanólico de *Cyperus rotundus* frente a *Streptococcus mutans* era de 225 y 450 mg/ml, respectivamente. Sin embargo, la concentración de 900 mg/ml representó una diferencia significativa con respecto a la clorhexidina al 0,2% y la penicilina de 500 mg. En nuestro estudio se utilizaron las concentraciones de 250, 500 y 1000 mg/ml del extracto etanólico de Cyperus rotundus sin encontrar efecto en los periodos de observación designados. Esto contrasta con lo obtenido por Yu *et al*. ^7^, quienes mostraron que, en concentraciones de 0,5, 1, 2 y 4 mg/ml de extracto etanólico del túberculo del *Cyperus rotundus* ya se producía inhibición del crecimiento para el *Streptococcus mutans*. 

La discrepancia entre nuestro estudio y los antecedentes puede originarse en la variabilidad de la planta y el método utilizado para reconocer el efecto inhibitorio. Todos los estudios previos donde se destaca su efectividad frente al *Streptococcus mutans* han sido realizados en otros países, lo que sugiere que las condiciones climatológicas locales pueden haber interferido con las propiedades antibacterianas de la planta. 

Una de las principales limitaciones de esta investigación es que se realizó durante la pandemia de COVID-19 y, por ello, fue mucho más difícil la disponibilidad de los materiales y laboratorios, además de los inconvenientes para conseguir muestras de pacientes. Fue por ello que se empleó una cepa ATCC, la cual es un cultivo puro estandarizado, con la desventaja de ser más susceptible a los agentes antimicrobianos en comparación con las cepas aisladas de pacientes. 

En la actualidad, existen pocos trabajos publicados con respecto a las propiedades y el efecto antibacteriano y antifúngico del extracto etanólico del *Cyperus rotundus* de origen peruano, siendo este, a nuestro conocimiento, el primero que evalúa su efecto sobre microrganismos orales. Aun cuando los resultados obtenidos no hayan sido favorables para las tres concentraciones del extracto, se recomienda continuar con la línea de investigación sobre plantas medicinales, especialmente sobre el *Cyperus rotundus*, y así complementar el conocimiento y encontrar la razón por la cual la variedad peruana de esta planta no presentó efectos inhibitorios sobre la bacteria estudiada. Se pueden ampliar estudios acerca de las características químicas, las estructurales, los mecanismos de acción y el principio activo. Asimismo, se recomienda que, en futuras investigaciones, se apliquen otros procedimientos más eficaces, como el método de pozos modificados de agar.

## CONCLUSIONES

El presente estudio demostró que el extracto etanólico del *Cyperus rotundus* (Cajamarca, Contumazá), en concentraciones de 250, 500 y 1000 mg/ml, no presenta actividad inhibitoria frente a *Streptococcus mutans* (ATCC®25175^TM^) a las 48 horas. Se recomienda profundizar en el análisis químico de los componentes de esta planta y explorar otros métodos de extracción para verificar su acción bacteriostática en diversos microorganismos orales y no orales. 

## Fuente de financiamiento:

Financiado por el Fondo Semilla para proyectos de investigación docente de la Universidad Científica del Sur (2019)
